# Biomimetic Nanocarrier Targeting Drug(s) to Upstream-Receptor Mechanisms in Dementia: Focusing on Linking Pathogenic Cascades

**DOI:** 10.3390/biomimetics5010011

**Published:** 2020-03-20

**Authors:** Joseph S. D’Arrigo

**Affiliations:** Cavitation-Control Technology Inc., Farmington, CT 06032, USA; cavcon@ntplx.net

**Keywords:** Alzheimer’s disease, calcium dyshomeostasis, dementia, drug targeting, endothelial dysfunction, inflammation, nanocarrier, oxidative stress

## Abstract

Past published studies have already documented that, subsequent to the intravenous injection of colloidal lipid nanocarriers, apolipoprotein (apo)A-I is adsorbed from the blood onto the nanoparticle surface. The adsorbed apoA-I mediates the interaction of the nanoparticle with scavenger receptors on the blood–brain barrier (BBB), followed by receptor-mediated endocytosis and subsequent transcytosis across the BBB. By incorporating the appropriate drug(s) into biomimetic (lipid cubic phase) nanocarriers, one obtains a multitasking combination therapeutic which targets certain cell-surface scavenger receptors, mainly class B type I (i.e., SR-BI), and crosses the BBB. Documented similarities in lipid composition between naturally occurring high-density lipoproteins (HDL) and the artificial biomimetic (nanoemulsion) nanocarrier particles can partially simulate or mimic the known heterogeneity (i.e., subpopulations or subspecies) of HDL particles. Such biomedical application of colloidal drug-nanocarriers can potentially be extended to the treatment of complex medical disorders like dementia. The risk factors for dementia trigger widespread inflammation and oxidative stress; these two processes involve pathophysiological cascades which lead to neuronal Ca^2+^ increase, neurodegeneration, gradual cognitive/memory decline, and eventually (late-onset) dementia. In particular, more recent research indicates that chronic inflammatory stimulus in the gut may induce (e.g., via serum amyloid A (SAA)) the release of proinflammatory cytokines. Hence, an effective preventive and therapeutic strategy could be based upon drug targeting toward a major SAA receptor responsible for the SAA-mediated cell signaling events leading to cognitive decline and eventually Alzheimer’s disease or (late-onset) dementia.

## 1. Introduction

Microvascular endothelial dysfunction, due to cerebrovascular risk factors, precedes cognitive decline in Alzheimer’s disease and contributes to its pathogenesis (see [1,2] for reviews). These risk factors (e.g., hypertension, diabetes, obesity, atherosclerosis, smoking, aging) trigger widespread inflammation and oxidative stress; these two processes involve pathophysiological cascades which lead to neuronal Ca^2+^ increase, neurodegeneration, gradual cognitive/memory decline, and eventually Alzheimer’s disease or (late-onset) dementia [3].

## 2. Endothelial Dysfunction, and Targeted Treatment for Early Dementia

It has been reported repeatedly that *endothelial* modulation and repair is feasible by pharmacological targeting [1,2,4,5,6,7,8,9,10] of the *SR-BI* receptors (i.e., “scavenger receptor class B, type I”) [10]. As the detailed review by Mahringer et al. [11] points out, the blood-brain barrier (BBB) is equipped with several endocytic receptors at the luminal surface (i.e., the capillary endothelial membrane), including SR-BI. Recently, Fung et al. [12] specifically found that SR-BI mediates the uptake and transcytosis of high-density lipoproteins (HDL) across brain microvascular endothelial cells (i.e., across the BBB). Since SR-BI has already been identified as a major receptor for HDL (with their major apolipoprotein (apo)A-I), as well as for the recently reviewed [1,2] “lipid-coated microbubble/nanoparticle-derived” (LCM/ND) nanoemulsion (see below), this multitasking lipid nanoemulsion can arguably serve as a targeted, apoA-I-based, (SR-BI mediated) therapeutic agent for common (late-onset) dementias [13,14,15]. 

**Figure 1:**
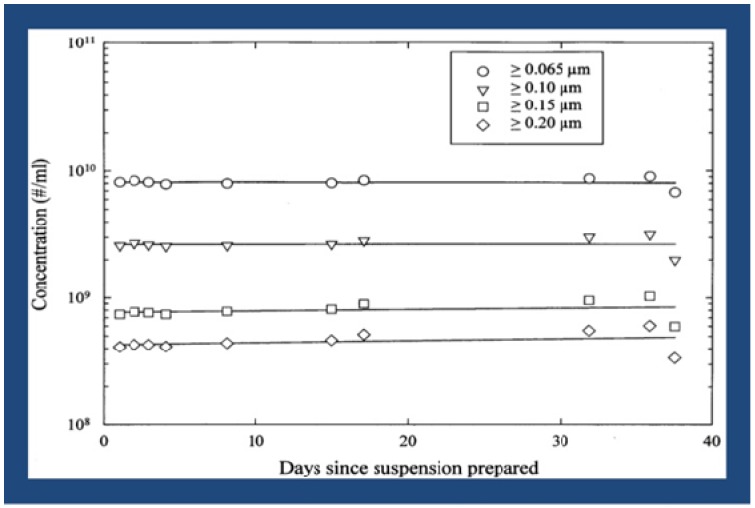
Lipid-coated microbubble/nanoparticle-derived (LCM/ND) nanoemulsion stability over time [2].

This targeted-delivery-approach, using the proposed LCM/ND lipid nanoemulsion for treating the more common (late-onset) dementias, receives added impetus from continual findings of cerebrovascular pathology [1,16,17,18,19,20,21,22,23,24,25,26] and an apparent *endothelium* dysfunction [2,15,19,22,27,28,29,30,31,32,33] in both Alzheimer’s disease and its major risk factors [1,2,26,27,28,29,30,31,32,33,34,35,36,37,38]. By incorporating drug molecules into the LCM/ND lipid nanoemulsion type (yielding particle sizes mostly < 0.1 μm in diameter —see Figure 1), known to be a successful drug carrier [39,40], one is likely to obtain a multitasking combination therapeutic capable of targeting cell-surface SR-BI. This (intravenous) combination therapeutic would make it possible for various cell types, all potentially implicated in Alzheimer’s disease [1,2], to be simultaneously sought out and better reached for the localized drug treatment of brain tissue in vivo [39,40].

## 3. Colloidal Nanocarrier Formation, and Targeting via Lipid Cubic Phases

In this particular targeted-delivery approach, the self-assembled colloidal “nanocarrier” structure itself (upon the intravenous injection of LCM/ND lipid nanoemulsion) is apparently successfully utilized as the “active” targeting ligand—which is directed via (adsorption of) plasma lipoproteins (including notably apoA-I) toward the appropriate endocytic receptors on the target-cell surface [39].

Previous reports concerning colloidal nanocarriers (e.g., [41,42]) do not fully explain how various (biobased) lipids, and their mixtures, are able to reliably form self-assembled non-lamellar nanostructures (i.e., lipid cubic phases)—which, in turn, have been observed to serve as colloidally stable nanocarriers for drug(s) in excess water (e.g., in blood plasma). The answer to this fundamental question resides in the physicochemical tendency of these biobased lipids to adopt a non-lamellar inverse topology [43]. This special tendency of these surface-active lipids is itself a function of lipid head-group hydration, acyl chain length, and cholesterol content (cf. below). In general, by increasing the average negative curvature of the lipid/water interface (e.g., by means of the water concentration or temperature), inverse-topology liquid–crystalline lipid phases (viz. different from lamellar) can be obtained—namely, inverse bicontinuous cubic phases, inverse hexagonal phases, or inverse (discontinuous) micellar cubic phases [44]. Moreover, Pouzot et al. have asserted that there is actual consensus on the fact that the formation of an (*Fd3m*) micellar cubic phase is promoted in systems where lipids have a negative preferred curvature, which is practically realized when long alkyl hydrophobic tails are associated with weakly hydrated, hydrophilic head groups ([44]; cf. [45]).

Notice that this actual concensus that amphiphilic lipids with weakly hydrated, hydrophilic head groups serve to promote the formation of an *Fd3m* cubic phase (also known as phase Q^227^) is particularly relevant to the earlier-described [42] LCM/ND nanoemulsion formulation(s): Specifically, the saturated glycerides and cholesterol (and its ester derivatives), which together compose the basic Filmix® (LCM/ND) nanoemulsion formulation [39], are all non-ionic and therefore each amphiphilic lipid in such a lipid mixture would only have a weakly hydrated, hydrophilic head group. Consequently, the above facts considered together support the earlier provisional conclusion that the dispersed *Fd3m* micellar cubic phase represents the most probable or preferred lipid polymorphic form adopted by the particles in the LCM/ND nanoemulsions [42,46].

In summary, the dispersed lipid particles of LCM/ND nanoemulsions very likely represent liquid–crystalline inverse-topology nanocarriers, i.e., dispersed lipid cubic phases (cf. [39]).

## 4. Cardiovascular Risk Factors, Inflammation, Oxidative Stress, Calcium Dyshomeostasis, and SR-BI

The cardiovascular risk factors for dementia induce brain tissue hypoxia, leading to endothelial cell activation. The result is the production/release of reactive oxygen species (ROS) and proinflammatory proteins, which together trigger widespread inflammation and oxidative stress—both of which can lead to BBB disruption [47]. (Note that inflammation is intimately associated with oxidative stress in Alzheimer’s disease. The redox status modulates inflammatory factors involvement in signaling processes, which are critical mediators of oxidative stress and neuroinflammation, causing neurodegeneration. The resultant cellular damage promotes further neuroinflammation in the Alzheimer’s-disease brain [48].) These pathological cascades lead to a neuronal Ca^2+^ increase, neurodegeneration, gradual cognitive/memory decline, and eventually Alzheimer’s disease [3].

It is believed by many researchers that enhanced calcium load may be brought about by extracellular accumulation of amyloid-β (Aβ) in the brain. Such studies have laid the foundation for the popular idea that Aβ peptides (39–42 amino acid molecules) are, in part, toxic to brain tissue because they form aberrant ion channels in cellular membranes and thereby disrupt Ca^2+^ homeostasis in brain tissue and increase intracellular Ca^2+^ [49,50]. Historical support for the above amyloid-β ion channel hypothesis, or so-called “calcium hypothesis”, has also been observed at the clinical level [51]. A good correlation exists between early cognitive impairment and levels of soluble forms of Aβ in the brain (but not the (insoluble) amyloid deposits or plaques at autopsy) [52]. Moreover, a recent biochemical study [53] of the two major Aβ variants, Aβ(1–40) and Aβ(1–42), has shown that: 1) Aβ(1–40) aggregated into amyloid fibrils; 2) contrariwise, Aβ(1–42) assembled into oligomers that inserted into membranes (i.e., artificial bilayers and/or biological membranes excised from cells of neuronal origin) as well-defined pores. (These amyloid pores adopted characteristics of a β-barrel arrangement.) Because Aβ(1–42), relative to Aβ(1–40), has a more prominent role in Alzheimer’s disease, the higher propensity of Aβ(1–42) to form β-barrel pore-forming oligomers is an indication of their importance in Alzheimer’s disease [53]. Furthermore, ion channel conductance results suggested that Aβ(1–42) oligomers, but not monomers and fibrils, formed pore structures. The authors concluded that their findings demonstrate that only Aβ(1–42) contains unique structural features that facilitate membrane insertion and channel formation, now aligning ion channel formation with the neurotoxic effect of Aβ(1–42) compared to Aβ(1–40) in Alzheimer’s disease [53]. (In addition, tea polyphenols have been repeatedly reported (e.g., [54]) to protect cells from Aβ-mediated neurotoxicity, by dose-dependently inhibiting the formation of Aβ aggregates (e.g., from fresh Aβ(1–42) peptides), through the destabilization of preformed Aβ aggregates. These green tea polyphenols (regularly ingested worldwide via tea beverage consumption) are considered to be valuable, for the prevention and therapeutic treatment of Alzheimer’s disease, via the combined effect of inhibiting Aβ aggregate formation and protecting neurons from the toxicity (e.g., oxidative stress) induced by Aβ [54].)

Note too that, while this Section 4 began with an acknowledgement that the risk factors for dementia trigger widespread inflammation and oxidative stress (e.g., [3]), it is also true that these two processes can result in more biological effects than enhanced calcium load in brain tissue and neurodegeneration (cf. [55]). In fact, oxidative stress and inflammation each involve pathophysiological cascades associated with a wide range of pathologies and especially aging. However, these two processes/cascades are not always associated with biological damage. (For example, oxidative stress constitutes an important mechanism in many physiological processes, such as adaptations to physical exercise and cell signaling.) Yet, when oxidative stress and/or inflammation are dysregulated, their action is harmful [55]. (In this situation, one corresponding example [of many] occurs in Alzheimer’s disease, where growing evidence links the ROS-mediated damages with molecular targets including mitochondrial dynamics/function, autophagic pathways, and proteostasis balance [56].) Accordingly, Khalil et al. [57] found that Alzheimer’s disease impaired the interaction of HDL (and ApoA-I) with the SR-BI receptor, and their experimental results indicated that such patients had higher levels of *oxidative stress* [57]. The authors concluded that their clinical study provides evidence for the first time that the functionality of HDL is impaired in Alzheimer’s disease, and that this alteration may be caused by Alzheimer’s disease-associated oxidative stress and inflammation [57]. This conclusion is consistent with earlier work where SR-BI was identified on astrocytes and vascular smooth muscle cells in Alzheimer’s disease brain, and has been demonstrated to mediate the adhesion of microglia to aggregated Aβ (cf. [58]). Moreover, these authors further report that SR-BI mediates perivascular macrophage response, and regulates Aβ-related pathology and cerebral amyloid angiopathy, in an Alzheimer’s-disease mouse model [58].

## 5. Gut-Brain Axis, Serum Amyloid A (SAA) versus SR-BI Targeting, and Alzheimer’s Disease or (late-onset) Dementia

Particularly noteworthy is more recent research [59,60] indicating that chronic inflammatory stimulus in the gut may induce (e.g., via *serum amyloid A* (SAA)) the release of proinflammatory cytokines. At the same time, increased BBB permeability due to aging (or dysfunction), in turn, allows these proinflammatory cytokines to enter the brain, inducing glia reactivity [59,60]. These recent findings and various past studies indicate that inflammation plays an important role in the process of Aβ deposition and, therefore, the inhibition of inflammatory cascades may attenuate amyloidogenic processes—such as Alzheimer’s disease [61] (cf. [57,62]). Hence, an effective preventive and therapeutic strategy could be based upon targeting drug(s) toward a major SAA receptor responsible for the SAA-mediated cell signaling events leading to cognitive decline and eventually Alzheimer’s disease or (late-onset) dementia.

Specifically, earlier research [63] has already confirmed that SR-BI receptors (or its human ortholog CLA-1) function as cell-surface SAA receptors—which bind, internalize, and mediate SAA-induced proinflammatory effects (cf. [64]). However, Baranova et al. additionally report that (in cell culture) CLA-1/SR-BI ligands “efficiently compete” with SAA for CLA-1/SR-BI binding [63]. (For example, it has already been documented in the literature that both apoA-I and SAA are substrates for SR-BI, which indicates that SR-BI could mediate the transport of both proteins across the BBB (e.g., [65])). Not surprisingly, therefore, Robert et al. have recently asserted that many lines of evidence suggest a protective role for HDL and its major apolipoprotein (apo)A-I in Alzheimer’s disease [14]. Accordingly, a similar benefit (of “competitive binding” to SR-BI receptors) may well accompany the clinical intravenous use of the LCM/ND lipid nanoemulsion vehicle—which has already been repeatedly described in the peer-reviewed literature (based upon numerous in vivo animal studies) as a targeted, apoA-I-based, (SR-BI mediated) drug-delivery agent (see Section 2). Moreover, by incorporating drug molecules into the LCM/ND lipid nanoemulsion type, one is likely to obtain a multitasking “combination therapeutic” capable of targeting cell-surface SR-BI. This (intravenous) colloidal-nanocarrier therapeutic would make it possible for various cell types, all potentially implicated in Alzheimer’s disease [1,2] and/or (late-onset) dementia, to be simultaneously sought out and better reached for localized drug treatment of brain tissue in vivo [39,40].

## 6. Conclusions

Cerebrovascular risk factors trigger widespread inflammation and oxidative stress, both of which can lead to BBB disruption. These pathological cascades lead to neuronal (intracellular) Ca^2+^ increase, neurodegeneration, gradual cognitive/memory decline, and eventually Alzheimer’s disease. In particular, more recent research indicates that chronic inflammatory stimulus in the gut may induce (e.g., via serum amyloid A (SAA)) the release of proinflammatory cytokines. At the same time, increased BBB permeability due to aging and/or dysfunction, in turn, allows these proinflammatory cytokines to enter the brain, inducing glia reactivity. An effective preventive and therapeutic strategy could be based upon early (or even proactive) targeting of drug(s) toward a major SAA receptor responsible for the SAA-mediated cell signaling events leading to cognitive decline, and eventually Alzheimer’s disease or (late-onset) dementia.
